# Preoperative donor urinary UDP-Glc as an independent risk factor for delayed graft function

**DOI:** 10.3389/fimmu.2025.1545280

**Published:** 2025-03-17

**Authors:** Maolin Ma, Fei Han, Qianghua Leng, Xiaorong Chen, Zuofu Tang, Jinhua Zhang, You Luo, Yang Zhang, Zhengyu Huang, Ning Na

**Affiliations:** Organ Transplantation Research Institution, Division of Kidney Transplantation, Department of Surgery, The Third Affiliated Hospital of Sun Yat-sen University, Guangzhou, Guangdong, China

**Keywords:** kidney transplantation, delayed graft function, tissue damage, donor urinary UDP-Glc, predictive model

## Abstract

**Background:**

Expanded criteria donors (ECD) have the potential to greatly increase the donor organ pool but pose a higher risk of delayed graft function (DGF) post-transplantation. Uridine diphosphate-glucose (UDP-Glc) plays a significant role in extracellular signaling related to tissue damage and retains stability for detection. Donor urinary UDP-Glc level may be an appropriate and effective biomarker for predicting DGF.

**Methods:**

Recipients who underwent successful kidney transplantation, with corresponding collection of donor urine samples, between June 2023 and August 2024 were included. We measured preoperative donor urinary UDP-Glc levels and analyzed their correlation with graft recovery. The study was registered in the Clinical Trial Registry (no. NCT06707272).

**Results:**

Preoperative donor urinary UDP-Glc levels were different between immediated, slowed, and delayed graft function subgroups (7.23 vs. 9.04 vs. 10.13 ug/mL, *p <* 0.001). Donor urinary UDP-Glc level was an independent risk factor for DGF (odds ratio [OR] = 1.741, 95% confidence interval [CI]: 1.311–2.312, *p <* 0.001). Furthermore, donor urinary UDP-Glc showed a better predictive value for DGF (AUROC = 0.791, 95% CI: 0.707–0.875, *p <* 0.001), and combining donor urinary UDP-Glc and donor terminal serum creatinine improved the model predictive value for DGF (AUROC = 0.832, 95% CI: 0.756–0.908, Youden index = 0.56, sensitivity = 0.81, specificity = 0.75, PPV = 0.72, NPV = 0.83, *p <* 0.001). Additionally, the donor urinary UDP-Glc level was related to the recipient serum creatinine level at 1 month post-transplantation (r_s_ = 0.475, *p <* 0.001).

**Conclusions:**

Donor urinary UDP-Glc level is an independent risk factor for DGF and can provide surgeons with a novel strategy to predict DGF earlier and more accurately without invasive procedures.

**Clinical trial registration:**

https://clinicaltrials.gov, NCT06707272 identifier.

## 1 Introduction

Kidney transplantation is the preferred treatment for end-stage renal disease; however, organ shortage is the primary bottleneck restricting organ transplantation (1). Organs from Expanded Criteria Donors (ECD) have the potential to greatly increase the donor organ pool. However, they require careful selection and utilization. Deceased kidney donors often have a history of central nervous system fluid regulation disorders and inflammatory mediator release, which lead to hemodynamic instability, electrolyte and acid-base imbalances, and a higher risk of primary graft non-function or delayed graft function (DGF) in recipients post-transplantation (2, 3). Searching for appropriate and effective biomarkers to assess renal quality and predict DGF is a crucial issue in the field of kidney transplantation.

Uridine diphosphate-glucose (UDP-Glc) is a damage-associated molecular pattern molecule released by damaged cells (4). UDP-Glc is synthesized in the cytoplasm and transported into the lumen of the endoplasmic reticulum and Golgi apparatus, where it regulates the synthesis of carbohydrates and acts as a substrate to facilitate glycosylation reactions (5). UDP-Glc is an endogenous excitant of the G protein-coupled P2Y14 receptor (6). Additionally, this receptor in humans is expressed at high levels in adipose tissue, stomach, kidney, intestines, specific regions of the brain, skeletal muscle, spleen, lungs, and heart (7). Released UDP-Glc plays a significant role in extracellular signaling within these tissues (8). Activation of P2Y14 promotes neutrophil infiltration, the recruitment of monocytes and macrophages, and the activation of the immune response, ultimately leading to tissue damage (9). Intercalated cells in the collecting duct of the kidney act as sensors for UDP-Glc, and when the P2Y14 receptor on their apical membrane is activated, intercalated cells produce chemotactic cytokines that attract neutrophils to the kidney, causing kidney inflammation and the onset of acute kidney injury (AKI) (10, 11). Furthermore, the concentration of UDP-Glc is higher in the urine of patients with AKI than in those without it (12). UDP-Glc hydrolyzes slowly in the extracellular environment, which results in UDP-Glc being highly stable and easily detectable (5, 13). Therefore, donor urinary UDP-Glc can serve as an appropriate and effective biomarker to assess renal quality and predict DGF.

This study aimed to investigate the correlation between donor urinary UDP-Glc levels and post-transplant graft function in recipients. We hypothesized that the higher the donor urinary UDP-Glc level, the more severe the kidney damage, resulting in a higher probability of DGF. This will provide transplant surgeons with a novel strategy to predict DGF earlier and more accurately without invasive procedures, while also reducing medical costs.

## 2 Methods and materials

### 2.1 Study design

This observational clinical study included recipients who underwent successful kidney transplantation, with donor urine samples collected at our center between June 2023 and August 2024. The inclusion criteria were as follows: 1) donors aged between 15 and 75 years, 2) recipients aged between 15 and 75 years, 3) signature of an informed consent form, 4) organs from deceased donors without contraindications for organ donation, and 5) donors free from infectious diseases, including tuberculosis and HIV/AIDS. The exclusion criteria were as follows: 1) donors without urine samples, 2) recipients with multiple organ transplantation, 3) participation in other clinical trials, 4) recipients who died during the perioperative period, 5) recipients who experienced kidney transplant nephrectomy during the perioperative period, 6) recipients who did not have a follow-up visit at our center within 1 month after the first discharge, 7) donors or recipients under 15 years of age, and 8) other features considered unsuitable by researchers. Two researchers independently collected the clinical data of recipients and donors from the medical records and organ transplant response systems, respectively. A third researcher verified the data accuracy.

### 2.2 Ethical approval

This study adhered to the Consolidated Standards of Reporting Trials (CONSORT) guidelines, and was approved by the Ethics Committee of The Third Affiliated Hospital of Sun Yat-sen University (approval no. II2024-047-02). All the participants provided written informed consent. All organs utilized in the study were donated voluntarily, and none originated from executed prisoners. The study was registered in the Clinical Trial Registry (no. NCT06707272).

### 2.3 Outcomes of interest

In this study, we used serum creatinine (Scr) as the graft outcome based on follow-up visits to our center within the first month. DGF was defined as the need for dialysis treatment within the first week following transplantation or Scr ≥ 4.52 mg/dL at post-transplant one week (14). Immediated graft function (IGF) was defined as Scr < 2.50 mg/dL at post-transplant one week, and slowed graft function (SGF) was defined as Scr ≥ 2.50 mg/dL but < 4.52 mg/dL without the need for dialysis at post-transplant one week (15). Additionally, ECD were defined as donor age ≥ 60 years or age 50–59 years with the following criteria (≥ 2/3): terminal Scr ≥ 1.50 mg/dL, cerebrovascular accident, and history of hypertension (16).

### 2.4 Sample collection

Donor urine samples were collected from donors at the time of kidney procurement using sterile tubes, incubated at room temperature for 1 h, and subsequently centrifuged at 2–8 ℃ for about 20 min (2000–3000 rpm). The supernatant was carefully collected, distributed, and stored in -80 ℃. Prior to the test, samples were centrifuged again if precipitation occurred during storage. All time-zero biopsy samples of the donor kidneys were collected by the chief surgeon using 16-gauge needles prior to implantation. Frozen tissue sections were prepared and subjected to hematoxylin and eosin staining. Remuzzi pathology scores were evaluated by the pathologist on duty at our hospital.

### 2.5 Measurement of donor urinary UDP-Glc level

Donor urinary UDP-Glc was detected using an ELISA kit (MM-92704301; www.mmbio.cn). The detection process was as follows: 1) Sample dilution buffer (40 μL) was added to the wells of the coated ELISA plates, and 10 μL of the testing sample was added to each well. 2) ELISA reagents (50 μL) were added to each well. 3) The plate was sealed and incubated for 30 min at 37°C. 4) A diluted solution was prepared by mixing the concentrated detergent with distilled water at a 1:30 ratio. 5) The plate was unsealed, and the liquid was discarded. The plate was dried by swinging, and washing buffer was added to each well, kept for 30 s, and then removed. This was repeated five times, followed by drying via patting. 6) Color reagent A (50 μL) and color reagent B (50 μL) were added to each well, and the plate was incubated for 10 min at 37°C. 7) Stop solution (50 μL) was added to each well. 8) The optical density at 450 nm was measured within 15 min. Finally, the concentration of UDP-Glc was calculated by constructing a standard curve using the absorbance values obtained.

### 2.6 Statistical analysis

Continuous variables of normally distributed data (*p* > 0.05, Shapiro–Wilk test) of donors or recipients were reported as mean ± standard deviation, whereas median and interquartile range was used for skewed data (*p* < 0.05). Categorical variables regarding donor or recipient data were reported as counts and percentages (%). To analyze the differences in continuous variables for the clinical data of donors or recipients between the different groups, parametric analysis of variance (ANOVA) and Kruskal–Wallis tests were employed. Chi-square or Fisher’s exact tests were used to examine the differences in categorical variables of the clinical data of patients between the groups. Additionally, to identify relevant independent clinical risk factors for donors or recipients with DGF, we used multivariate logistic regression analysis with stepwise backward variable selection (significance threshold defined as *p* < 0.05). A receiver operating characteristic (ROC) curve was used to evaluate UDP-Glc and other biomarkers for predicting DGF, and optimal cutoff points were derived using the Youden index. Spearman’s correlation coefficient and multiple linear regression analyses were used to evaluate the correlation between UDP-Glc and Scr at 1 month post-transplantation. To control for Type I error inflation due to multiple testing, a Bonferroni correction was applied to adjust the significance level for the 3 independent comparisons. SPSS version 26.0 (IBM Corp., Armonk, NY, USA) was used for analysis, with *p* < 0.05 considered as statistically significant.

## 3 Results

### 3.1 Characteristics of donors and recipients

**Figure 1:**
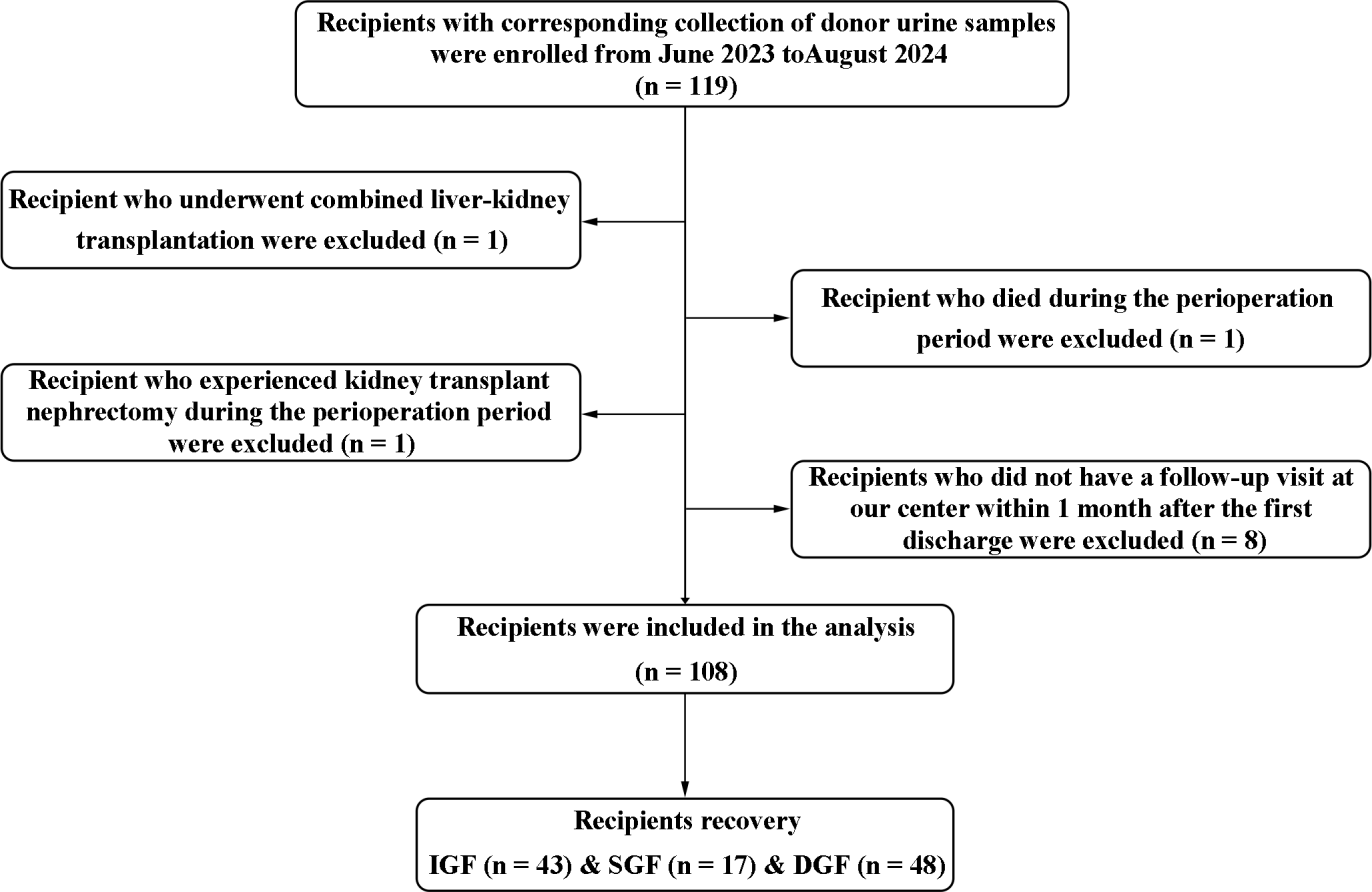
Flow diagram of the study for recipient enrollment.

From the study cohort (**Figure 1**), 119 recipients were enrolled, with corresponding collection of donor urine samples; Eleven recipients were excluded: one underwent combined liver-kidney transplantation, one died during the perioperative period, one experienced kidney transplant nephrectomy, and eight did not have a follow-up visit at our center within 1 month after the first discharge. Finally, the analysis included a total of 108 recipients. According to graft function, all patients were divided into three subgroups: IGF (n = 43), SGF (n = 17), and DGF (n = 48).

Donor characteristics are presented in **Table 1**. The mean donor age was 51.19 ± 10.64 years (49.21 ± 10.21 years in the IGF subgroup, 47.94 ± 8.32 years in the SGF subgroup, and 54.10 ± 11.18 years in the DGF subgroup; *p* = 0.034). The donor terminal Scr was 0.88 [0.72,1.50] mg/dL for the IGF subgroup, 2.12 [0.77,2.66] mg/dL for the SGF subgroup, and 2.01 [1.22,3.02] mg/dL for the DGF subgroup (*p <* 0.001). ECD was 8(18.60) for the IGF subgroup, 4(23.53) for the SGF subgroup, and 22(45.83) for the DGF subgroup (*p* = 0.014). The kidney donor profile index (KDPI) was 58.00 [47.00,75.00]% for the IGF subgroup, 70.00 [45.00,75.50]% for the SGF subgroup, and 76.50 [56.75,86.00]% for the DGF subgroup (*p =* 0.005). The donor urinary UDP-Glc was 7.23 [6.05,8.96] μg/mL for the IGF subgroup, 9.04 [8.23,10.95] μg/mL for the SGF subgroup, and 10.13 [8.64,11.32] μg/mL for the DGF subgroup (*p <* 0.001). No significant differences were observed in donor sex, donor body mass index (BMI), hypertension, cause of death, or Remuzzi pathology scores between the IGF, SGF, and DGF subgroups. The characteristics of the recipients are presented in **Table 2**. Pre-transplantation recipient features were not different between the three subgroups.

**Table 1:** Summary of characteristics in donors, stratified by recipients allograft function.

Characteristics	All (*N* = 108)	IGF (*N* = 43)	SGF (*N* = 17)	DGF (*N* = 48)	*P*-value
Age (years)	51.19 ± 10.64	49.21 ± 10.21	47.94 ± 8.32	54.10 ± 11.18	0.034
Male sex	86 (79.63)	33 (76.74)	14 (82.35)	39 (81.25)	0.810
BMI (kg/m^2^)	23.90 ± 2.58	23.80 ± 2.47	23.13 ± 1.89	24.25 ± 2.85	0.296
Hypertension	29 (26.85)	13 (30.23)	6 (35.29)	10 (20.83)	0.431
Cause of death					0.129
CVD	57 (52.78)	24 (55.81)	10 (58.82)	23 (47.92)	
Trauma	45 (41.67)	19 (44.19)	7 (41.18)	19 (39.58)	
Others	6 (5.55)	0 (0.00)	0 (0.00)	6 (12.50)	
Terminal Scr (mg/dL)	1.39 [0.84,2.44]	0.88 [0.72,1.50]	2.12 [0.77,2.66]	2.01 [1.22,3.02]	<0.001
Expanded criteria donor	34 (31.48)	8 (18.60)	4 (23.53)	22 (45.83)	0.014
KDPI (%)	71.50 [53.00,81.00]	58.00 [47.00,75.00]	70.00 [45.00,75.50]	76.50 [56.75,86.00]	0.005
Urinary UDP-Glc (ug/mL)	8.98 [7.36,10.20]	7.23 [6.05,8.96]	9.04 [8.23,10.95]	10.13 [8.64,11.32]	<0.001
Remuzzi pathology scores	3.00 [2.00,5.00]	2.00 [2.00,4.00]	2.00 [1.00,5.50]	4.00 [2.00,5.00]	0.180
Glomerular global sclerosis					0.085
0	57 (52.78)	25 (58.14)	10 (58.82)	22 (45.83)	
1	36 (33.33)	13 (30.23)	3 (17.65)	20 (41.67)	
2	9 (8.33)	1 (2.33)	3 (17.65)	5 (10.42)	
3	3 (2.78)	2 (4.65)	1 (5.88)	0 (0.00)	
Tubular atrophy					0.429
0	28 (25.93)	12 (27.91)	7 (41.18)	9 (18.75)	
1	71 (65.74)	28 (65.12)	10 (58.82)	33 (68.75)	
2	4 (3.70)	1 (2.33)	0 (0.00)	3 (6.25)	
3	2 (1.85)	0 (0.00)	0 (0.00)	2 (4.17)	
Interstitial fibrosis					0.532
0	31 (28.70)	14 (32.56)	7 (41.18)	10 (20.83)	
1	69 (63.89)	26 (60.47)	10 (58.82)	33 (68.75)	
2	3 (2.78)	1 (2.33)	0 (0.00)	2 (4.17)	
3	2 (1.85)	0 (0.00)	0 (0.00)	2 (4.17)	
Arterial and arteriolar narrowing					0.475
0	42 (38.89)	19 (44.19)	7 (41.18)	16 (33.33)	
1	33 (30.56)	10 (23.26)	5 (29.41)	18 (37.50)	
2	22 (20.37)	10 (23.26)	2 (11.76)	10 (20.83)	
3	8 (7.41)	2 (4.65)	3 (17.65)	3 (6.25)	
Kidney					0.841
Left	53 (49.07)	22 (51.16)	9 (52.94)	22 (45.83)	
Right	55 (50.93)	21 (48.84)	8 (47.06)	26 (54.17)	

The continuous variables were analyzed using the Shapiro-Wilk test. If P > 0.05, the data are expressed as the mean ± SD; otherwise, the data are expressed as the median [P25, P75]. The categorical variables are described using total numbers and percentages (%).

ANOVA or Kruskal–Wallis tests were used to analyze continuous variables and χ^2^ test or Fisher’s exact test were used to analyze categorical variables.

IGF, immediated graft function; SGF, slowed graft function; DGF, delayed graft function; BMI, body mass index; CVD, cerebrovascular disease; Scr, serum creatinine; KDPI, kidney donor profile index; UDP-Glc, uridine diphosphate-glucose; ANOVA, analysis of variance.

**Table 2:** Summary of characteristics in recipients, stratified by recipient allograft function.

Characteristics	All (*N* = 108)	IGF (*N* = 43)	SGF (*N* = 17)	DGF (*N* = 48)	*P*-value
Age (years)	44.44 ± 12.44	46.05 ± 12.40	38.71 ± 9.57	45.04 ± 13.01	0.108
Male sex	76 (70.37)	26 (60.47)	14 (82.35)	36 (75.00)	0.159
BMI (kg/m^2^)	22.75 [20.30,26.32]	21.66 [19.61,24.88]	25.56 [22.56,26.85]	23.18 [20.30,27.10]	0.080
Transplantation history					1.000
Yes	8 (7.41)	3 (6.98)	1 (5.88)	4 (8.33)	
No	100 (92.59)	40 (93.02)	16 (94.12)	44 (91.67)	
Cause of ESRD					0.966
GN	26 (24.07)	10 (23.26)	5 (29.41)	11 (22.92)	
DN	11 (10.19)	5 (11.63)	1 (5.88)	5 (10.42)	
HTN	5 (4.63)	3 (6.98)	0 (0.00)	2 (4.17)	
PKD	4 (3.70)	2 (4.65)	1 (5.88)	1 (2.08)	
Others	62 (57.41)	23 (53.49)	10 (58.82)	29 (60.42)	
Dialysis modality					0.643
HD	85 (78.70)	33 (76.74)	12 (70.59)	40 (83.33)	
PD	18 (16.67)	7 (16.28)	4 (23.53)	7 (14.58)	
No dialysis	5 (4.63)	3 (6.98)	1 (5.88)	1 (2.08)	
Dialysis vintage					0.683
0	5 (4.63)	3 (6.98)	1 (5.88)	1 (2.08)	
1∼6 months	18 (16.67)	9 (20.93)	2 (11.76)	7 (14.58)	
7∼12 months	27 (25.00)	8 (18.60)	6 (35.29)	13 (27.08)	
13∼36 months	41 (37.04)	17 (39.53)	7 (41.18)	17 (35.42)	
>36 months	17 (15.74)	6 (13.95)	1 (5.88)	10 (20.83)	
Number of HLA mismatches	5.00 [3.50,5.00]	4.00 [3.00,5.00]	4.00 [4.00,5.00]	5.00 [4.00,5.00]	0.073
WIT (min)	0.00 [0.00,0.00]	0.00 [0.00,0.00]	0.00 [0.00,0.00]	0.00 [0.00,0.00]	0.283
CIT (h)	7.98 [6.36,9.37]	8.47 [6.35,9.42]	7.15 [6.33,8.96]	7.85 [6.36,9.63]	0.737
PRA					0.410
0%	91 (84.26)	39 (90.70)	15 (88.24)	37 (77.08)	
1∼10%	15 (13.89)	4 (9.30)	2 (11.76)	9 (18.75)	
>10%	2 (1.85)	0 (0.00)	0 (0.00)	2 (4.17)	
CNI					0.374
Tacrolimus	100 (92.59)	38 (88.37)	16 (94.12)	46 (95.83)	
Cyclosporin	8 (7.41)	5 (11.63)	1 (5.88)	2 (4.17)	
MPA	108 (100.00)	43 (100.00)	17 (100.00)	48 (100.00)	1.000
Glucocorticoids	108 (100.00)	43 (100.00)	17 (100.00)	48 (100.00)	1.000
Renal Function (mg/dL)					
Scr, pre-transplantation	9.96 [7.89,11.69]	9.16 [7.30,11.66]	10.78 [8.00,13.49]	10.00 [8.35,11.99]	0.361
Scr, 3 days post-transplantation	5.50 [2.94,8.78]	2.32 [1.44,3.89]	6.91 [5.46,8.79]	8.51 [5.56,10.56]	<0.001
Scr, 1 week post-transplantation	3.54 [1.78,6.68]	1.46 [1.03,2.00]	3.52 [2.88,3.91]	6.81 [5.27,8.81]	<0.001
Scr, 2 weeks post-transplantation	2.30 [1.41,4.16]	1.35 [0.95,1.70]	2.34 [1.62,2.78]	4.34 [2.96,6.06]	<0.001
Scr, 1 month post-transplantation	1.65 [1.29,2.79]	1.23 [0.94,1.53]	1.89 [1.42,3.00]	2.63 [1.65,3.70]	<0.001

The continuous variables were analyzed using the Shapiro-Wilk test. If P > 0.05, the data are expressed as the mean ± SD; otherwise, the data are expressed as the median [P25, P75]. The categorical variables are described using total numbers and percentages (%).

ANOVA or Kruskal–Wallis tests were used to analyze continuous variables and χ^2^ test or Fisher’s exact test were used to analyze categorical variables.

IGF, immediated graft function; SGF, slowed graft function; DGF, delayed graft function; BMI, body mass index; ESRD, end-stage renal disease; GN, glomerulonephritis; DN, diabetic nephropathy; HTN, hypertensive nephropathy; PKD, polycystic kidney disease; HD, hemodialysis; PD, peritoneal dialysis; HLA, human leukocyte antigen; WIT, warm ischemia time; CIT, cold ischemia time; PRA, panel reaction antibody; CNI, calcineurin inhibitor; MPA, mycophenolic acid; Scr, serum creatinine; ANOVA, analysis of variance.

### 3.2 Distinction of donor urinary UDP-Glc levels among recipients with different graft function

**Figure 2:**
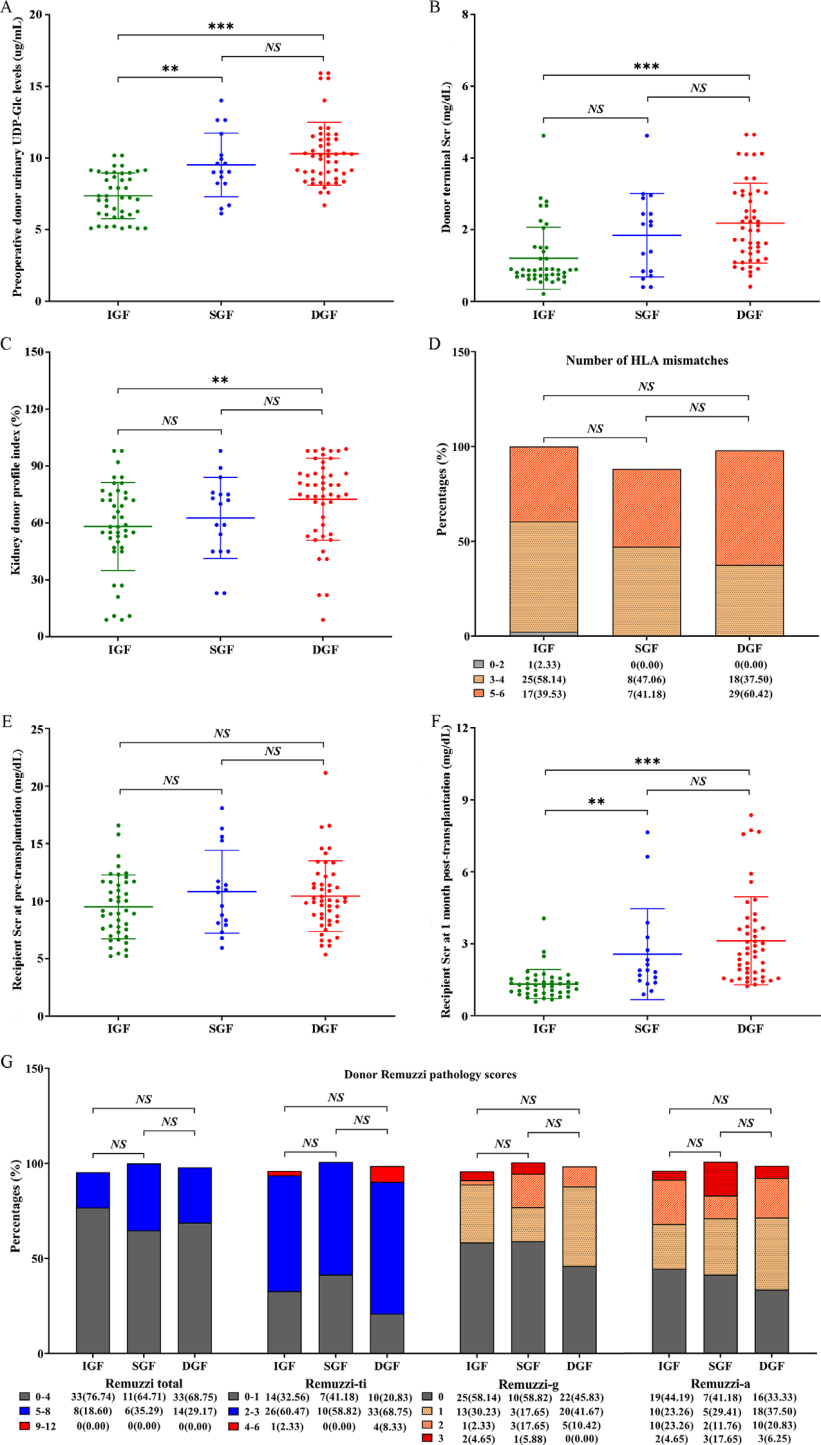
Distribution of several relevant clinical factors in the IGF, SGF, and DGF subgroups. **(A)** Preoperative donor urinary UDP-Glc levels; **(B)** donor terminal Scr; **(C)** kidney donor profile index; **(D)** number of HLA mismatches; **(E)** recipient Scr at pre-transplantation; **(F)** recipient Scr at 1 month post-transplantation; **(G)** donor Remuzzi pathology scores. IGF, immediated graft function; SGF, slowed graft function; DGF, delayed graft function; UDP-Glc, Uridine diphosphate glucose; Scr, serum creatinine; HLA, human leukocyte antigen; g, glomerular global sclerosis; ti, tubular atrophy and interstitial fibrosis; a, arterial and arteriolar narrowing. *NS*, non-significant; **p < 0.01; ***p < 0.001.

The donor urinary UDP-Glc was higher in the DGF subgroup (n = 48) than in the non-DGF subgroup (n = 60) (10.13 [8.64,11.32] μg/mL vs. 8.08 [6.27,9.08] μg/mL, *p <* 0.001), and the differences in other clinical characteristics between the two subgroups are shown in **Supplementary Figure S1**. Subsequently, we found that donor urinary UDP-Glc in the IGF subgroup was significantly lower compared to the SGF (7.23 [6.05,8.96] μg/mL vs. 9.04 [8.23,10.95] μg/mL, *p =* 0.003) and DGF (7.23 [6.05, 8.96] μg/mL vs. 10.13 [8.64,11.32] μg/mL, *p <* 0.001) subgroups, but there were no significant differences between the SGF and DGF subgroups (*P =* 0.760; **Figure 2A**). Additionally, compared to the DGF subgroup, the IGF subgroup had a lower donor terminal Scr level (0.88 [0.72,1.50] mg/dL vs. 2.01 [1.22,3.02] mg/dL, *p <* 0.001; **Figure 2B**) and a lower KDPI (58.00 [47.00,75.00]% vs. 76.50 [56.75,86.00]%, *p =* 0.005; **Figure 2C**). However, no significant differences were observed in the number of human leukocyte antigen (HLA) mismatches, recipient Scr at pre-transplantation, or donor Remuzzi pathology scores (including Remuzzi-total, Remuzzi-g, Remuzzi-ti, and Remuzzi-a scores) between the three subgroups (**Figures 2D, E, G**; **Table 1**). Finally, we found that recipients in the IGF subgroup had a lower Scr at 1 month post-transplantation compared to those in the SGF subgroup (1.23 [0.94,1.53] mg/dL vs. 1.89 [1.42,3.00] mg/dL, *p* = 0.001) and DGF subgroup (1.23 [0.94,1.53] mg/dL vs. 2.63 [1.65,3.70] mg/dL, *p <* 0.001; **Figure 2F**).

### 3.3 Donor urinary UDP-Glc as an independent risk factor for DGF

In total, 48/108 (44.44) recipients had DGF. We analyzed the correlation between DGF and the relevant clinical parameters of donors and recipients (**Table 3**). Firstly, based on univariate logistic regression analyses, we found that donor urinary UDP-Glc (odds ratio [OR] = 1.769, 95% confidence interval [CI]: 1.366–2.291, *p <* 0.001), donor age (OR = 1.053, 95% CI: 1.011–1.097, *p* = 0.014), donor terminal Scr (OR = 2.045, 95% CI: 1.366–3.061, *p* = 0.001), ECD (OR = 3.385, 95% CI: 1.447–7.919, *p* = 0.005), KDPI (OR = 1.028, 95% CI: 1.009–1.048, *p* = 0.005), and panel reactive antibody levels (OR = 2.695, 95% CI: 1.004–7.238, *p* = 0.049) were significantly correlated with DGF. Subsequently, multivariate logistic regression analyses showed that donor urinary UDP-Glc (OR = 1.741, 95% CI: 1.311–2.312, *p <* 0.001), donor terminal Scr (OR = 1.684, 95% CI: 1.061–2.674, *p* = 0.027), and ECD (OR = 3.091, 95% CI: 1.075–8.887, *p* = 0.036) were independent risk factors for DGF.

**Table 3:** Univariate and multivariate logistic regression analyses for predicting delayed graft function.

	Univariate			Multivariate		
	OR	95% CI	*P*-value	OR	95% CI	*P*-value
Donor age (years)	1.053	1.011-1.097	0.014			
Donor sex (male)	1.199	0.464-3.099	0.709			
Donor BMI (kg/m^2^)	1.103	0.948-1.282	0.204			
Donor terminal Scr (mg/dL)	2.045	1.366-3.061	0.001	1.684	1.061-2.674	0.027
Expanded criteria donor	3.385	1.447-7.919	0.005	3.091	1.075-8.887	0.036
KDPI (%)	1.028	1.009-1.048	0.005			
Donor Remuzzi pathology scores	1.176	0.969-1.426	0.100			
Donor urinary UDP-Glc (ug/mL)	1.769	1.366-2.291	<0.001	1.741	1.311-2.312	<0.001
Cold ischemia time (h)	1.072	0.934-1.229	0.322			
Recipient age (years)	1.007	0.977-1.038	0.654			
Recipient sex (male)	1.500	0.644-3.494	0.347			
Recipient BMI (kg/m^2^)	1.059	0.973-1.153	0.188			
Dialysis vintage	1.261	0.880-1.806	0.206			
Transplantation history	1.273	0.301-5.377	0.743			
Number of HLA mismatches	1.490	0.996-2.229	0.052			
PRA	2.695	1.004-7.238	0.049			

The multivariate logistic regression analysis was performed using a backward selection procedure. The probability (Wald statistic) for the stepwise method was set at 0.05 for entry and 0.10 for removal.

OR, odds ratio; CI, confidence interval; BMI, body mass index; Scr, serum creatinine; KDPI, kidney donor profile index; UDP-Glc, uridine diphosphate-glucose; HLA, human leukocyte antigen; PRA, panel reactive antibody.

### 3.4 Predictive value of donor urinary UDP-Glc for DGF

**Figure 3:**
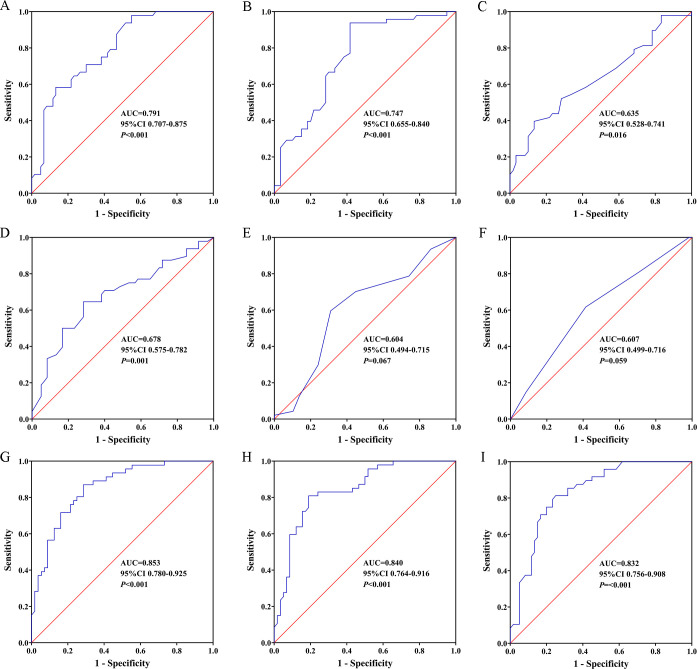
Predictive value of donor urinary UDP-Glc for DGF. Receiver operating characteristic (ROC) curves: **(A)** donor urinary UDP-Glc, **(B)** donor terminal Scr, **(C)** donor age, **(D)** kidney donor profile index, **(E)** donor Remuzzi pathology scores, **(F)** number of HLA mismatches, **(G)** predictive model including A+B+C+D+E+F, **(H)** predictive model including A+B+F, **(I)** predictive model including A+B. AUC, area under curve; CI, confidence interval; UDP-Glc, Uridine diphosphate glucose; DGF, delayed graft function.

ROC curve analysis was employed to evaluate the predictive value of relevant clinical parameters for DGF (**Figure 3**), and the Youden index, sensitivity, specificity, positive predictive value (PPV), and negative predictive value (NPV) at the optimal cut-off point are listed in **Table 4**. Firstly, the area under the ROC curve (AUROC) of the (A) donor urinary UDP-Glc, (B) donor terminal Scr, (C) donor age, (D) KDPI, (E) donor Remuzzi pathology scores, and (F) number of human leukocyte antigen (HLA) mismatches in predicting DGF were 0.791 (95% CI: 0.707–0.875, *p <* 0.001; **Figure 3A**), 0.747 (95% CI: 0.655–0.840, *p <* 0.001; **Figure 3B**), 0.635 (95% CI: 0.528–0.741, *p* = 0.016; **Figure 3C**), 0.678 (95% CI: 0.575–0.782, *p* = 0.001; **Figure 3D**), 0.604 (95% CI: 0.494–0.715, *p* = 0.067; **Figure 3E**), and 0.607 (95% CI: 0.499–0.716, *p* = 0.059; **Figure 3F**), respectively. Donor urinary UDP-Glc showed the best AUROC value for DGF compared to the other clinical factors. Based on the above analysis, we developed several predictive models. The model that included A+B+C+D+E+F improved the AUROC to 0.853 (95% CI: 0.780–0.925, Youden index = 0.58, sensitivity = 0.87, specificity = 0.71, PPV = 0.71, NPV = 0.88, *p <* 0.001; **Figure 3G**), the prediction formula was as follows: y = 0.567×donor urinary UDP-Glc + 0.422×donor terminal Scr + 0.049×donor terminal Scr + 0.008×KDPI + 0.178×donor Remuzzi pathology scores + 0.544×number of HLA mismatches - 12.193. The model that included A+B+F improved the AUROC to 0.840 (95% CI: 0.764–0.916, Youden index = 0.62, sensitivity = 0.81, specificity = 0.81, PPV = 0.78, NPV = 0.84, *p <* 0.001; **Figure 3H**), the prediction formula was as follows: y = 0.538×donor urinary UDP-Glc + 0.546×donor terminal Scr + 0.406×number of HLA mismatches - 7.809. The model that included A+B improved the AUROC to 0.832 (95% CI: 0.756–0.908, Youden index = 0.56, sensitivity = 0.81, specificity = 0.75, PPV = 0.72, NPV = 0.83, *p <* 0.001; **Figure 3I**), the prediction formula was as follows: y = 0.552×donor urinary UDP-Glc + 0.586×donor terminal Scr - 6.245.

**Table 4:** Sensitivity, specificity, and predictive values for predicting delayed graft function at optimum cut-off value of donor and recipient characteristic.

Characteristics	Cut-off value	Sensitivity	Specificity	PPV	NPV	Youden index	AUC (95%CI)
A-Donor urinary UDP-Glc (ug/mL)	9.67	0.58	0.87	0.78	0.72	0.45	0.791 (0.707-0.875)
B-Donor terminal Scr (mg/dL)	0.91	0.94	0.58	0.64	0.92	0.52	0.747 (0.655-0.840)
C-Donor age (years)	57.50	0.40	0.87	0.70	0.64	0.26	0.635 (0.528-0.741)
D-KDPI (%)	73.50	0.65	0.72	0.65	0.72	0.36	0.678 (0.575-0.782)
E-Donor Remuzzi pathology scores	3.50	0.60	0.69	0.60	0.68	0.29	0.604 (0.494-0.715)
F-Number of HLA mismatches	4.50	0.62	0.59	0.55	0.66	0.20	0.607 (0.499-0.716)
G-Model including A+B+C+D+E+F	0.37	0.87	0.71	0.71	0.88	0.58	0.853 (0.780-0.925)
H-Model including A+B+F	0.46	0.81	0.81	0.78	0.84	0.62	0.840 (0.764-0.916)
I-Model including A+B	0.44	0.81	0.75	0.72	0.83	0.56	0.832 (0.756-0.908)

PPV, positive predictive value; NPV, negative predictive value; AUC, area under curve; CI, confidence interval; UDP-Glc, uridine diphosphate-glucose; Scr, serum creatinine; KDPI, kidney donor profile index; HLA, human leukocyte antigen.

### 3.5 Donor urinary UDP-Glc is related to recipient Scr at 1 month post-transplantation

**Figure 4:**
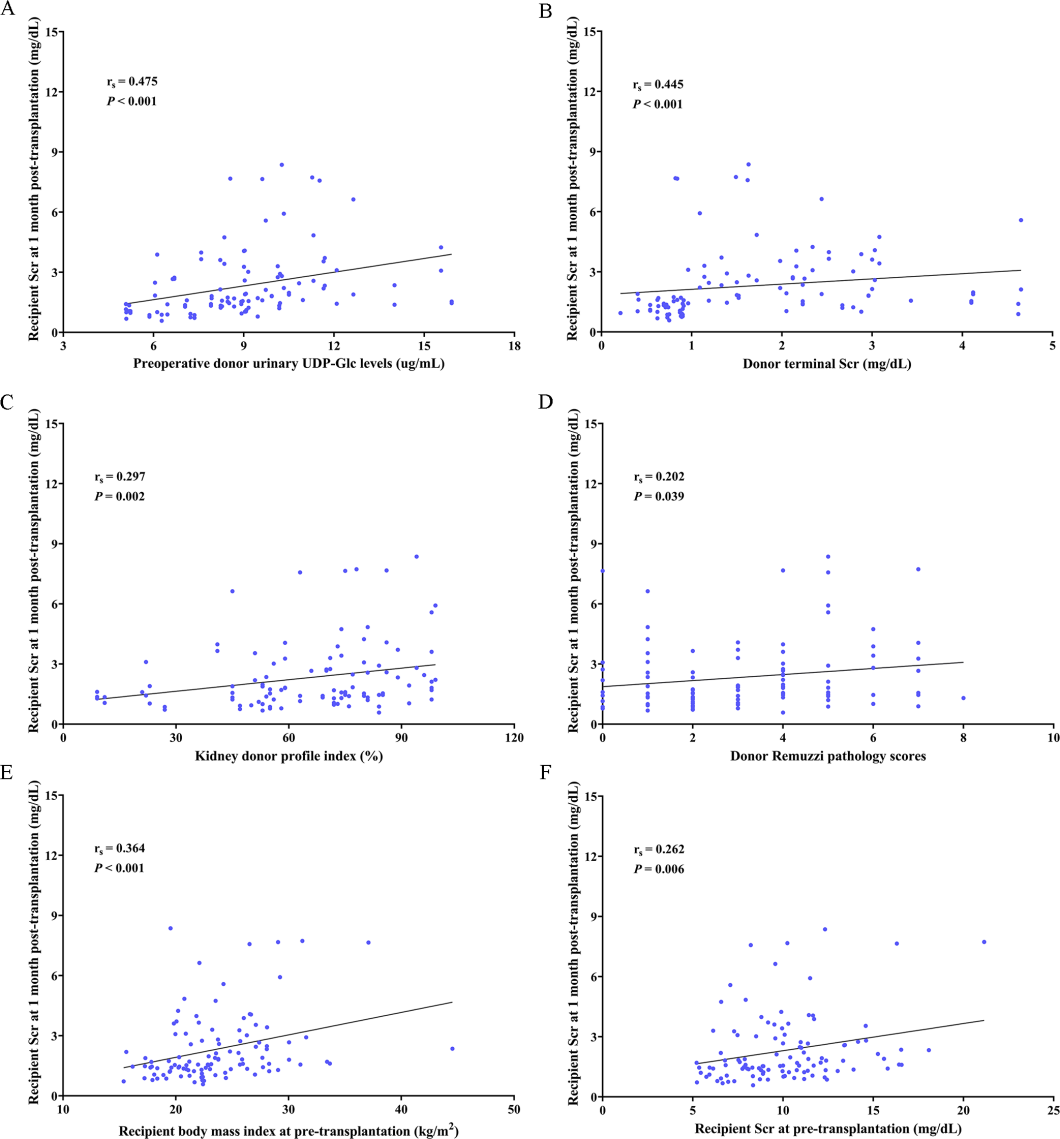
Donor urinary UDP-Glc is related to the recipient Scr at 1 month post-transplantation. Spearman’s correlation between **(A)** preoperative donor urinary UDP-Glc levels, **(B)** donor terminal Scr, **(C)** kidney donor profile index, **(D)** donor Remuzzi pathology scores, **(E)** recipient body mass index at pre-transplantation, and **(F)** recipient Scr at pre-transplantation and recipient Scr at 1 month post-transplantation. UDP-Glc, Uridine diphosphate glucose; Scr, serum creatinine.

We analyzed the correlation between recipient Scr at 1 month post-transplantation and the relevant clinical parameters of donors and recipients (**Table 5**). Based on the univariate linear regression analyses, we found that donor urinary UDP-Glc (β coefficient = 0.324, 95% CI: 0.100–0.357, *p* = 0.001), KDPI (β coefficient = 0.262, 95% CI: 0.006–0.033, *p* = 0.006), male recipients (β coefficient = 0.248, 95% CI: 0.228–1.601, *p* = 0.010), and recipient BMI (β coefficient = 0.307, 95% CI: 0.045–0.179, *p* = 0.001) were significantly related to recipient Scr at 1 month post-transplantation. Subsequently, according to multivariate linear regression analyses, we showed that donor urinary UDP-Glc (β coefficient = 0.306, 95% CI: 0.096–0.347, *p* = 0.001), donor Remuzzi pathology scores (β coefficient = 0.195, 95% CI: 0.018–0.306, *p* = 0.028), and recipient BMI (β coefficient = 0.303, 95% CI: 0.048–0.178, *p* = 0.001) were correlated with recipient Scr at 1 month post-transplantation. Additionally, we concluded that donor urinary UDP-Glc (r_s_ = 0.475, *p <* 0.001; **Figure 4A**), donor terminal Scr (r_s_ = 0.445, *P <* 0.001; **Figure 4B**), KDPI (r_s_ = 0.297, *p* = 0.002; **Figure 4C**), donor Remuzzi pathology scores (r_s_ = 0.202, *p* = 0.039; **Figure 4D**), recipient BMI (r_s_ = 0.364, *p <* 0.001; **Figure 4E**), and recipient Scr at pre-transplantation (r_s_ = 0.262, *p* = 0.006; **Figure 4F**) were significantly related to recipient Scr at 1 month post-transplantation by Spearman’s correlation coefficient analyses.

**Table 5:** Univariate and multivariate linear regression analyses for predicting 1 month graft function.

	Univariate			Multivariate		
	β coefficient	95% CI	*P*-value	β coefficient	95% CI	*P*-value
Donor age (years)	0.149	-0.007 to 0.054	0.123			
Donor sex (male)	-0.013	-0.858 to 0.749	0.894			
Donor BMI (kg/m^2^)	0.021	-0.112 to 0.140	0.828			
Donor terminal Scr (mg/dL)	0.173	-0.026 to 0.548	0.074			
Expanded criteria donor	0.150	-0.146 to 1.233	0.121			
KDPI (%)	0.262	0.006 to 0.033	0.006			
Donor Remuzzi pathology scores	0.183	-0.008 to 0.311	0.062	0.195	0.018 to 0.306	0.028
Donor urinary UDP-Glc (ug/mL)	0.324	0.100 to 0.357	0.001	0.306	0.096 to 0.347	0.001
Cold ischemia time (h)	0.013	-0.108 to 0.123	0.895			
Recipient age (years)	0.110	-0.011 to 0.041	0.258			
Recipient sex (male)	0.248	0.228 to 1.601	0.010			
Recipient BMI (kg/m^2^)	0.307	0.045 to 0.179	0.001	0.303	0.048 to 0.178	0.001
Dialysis vintage	-0.113	-0.472 to 0.122	0.246			
Transplantation history	0.028	-1.059 to 1.412	0.777			
Number of HLA mismatches	-0.067	-0.446 to 0.218	0.497			
PRA	0.081	-0.438 to 1.074	0.406			

The multivariate logistic regression analysis was performed using a backward selection procedure. The probability (Wald statistic) for the stepwise method was set at 0.05 for entry and 0.10 for removal.

CI, confidence interval; BMI, body mass index; Scr, serum creatinine; KDPI, kidney donor profile index; UDP-Glc, uridine diphosphate-glucose; HLA, human leukocyte antigen; PRA, panel reaction antibody.

## 4 Discussion

In this study, we evaluated the potential of donor urinary UDP-Glc as a biomarker and found it to be an independent risk factor for DGF. Furthermore, the predictive value of donor urinary UDP-Glc for DGF was higher than that of other biomarkers. Additionally, donor urinary UDP-Glc levels were positively correlated with recipient Scr levels at 1 month after transplantation.

DGF is a common complication that can have detrimental effects on graft survival, cause acute rejection, increase hospital stay, and affect renal function (17, 18). AKI plays an important role in DGF, and several researchers and surgeons believe that DGF is a form of AKI (19). UDP-Glc is a key component of damage-associated molecular patterns and is released from injured cells. When kidney injury occurs, UDP-Glc reaches the lumen of the collecting duct, binds and activates P2Y14 located on the apical membrane of intercalated cells, and triggers renal inflammation, leading to proximal tubule injury and causing additional damage (20). Lazarowski et al. demonstrated that urinary UDP-Glc levels were higher in patients with AKI than in those without it (12). Our results also show that patients with DGF were associated with higher levels of donor urinary UDP-Glc.

Organ shortage limits the widespread use of kidney transplantation; ECD is used to reduce the shortage of kidneys for transplantation; however, it contributes to a higher risk of DGF, and we anticipate that the incidence of DGF is expected to increase in the coming years (21–24). Additionally, DGF is an independent risk factor for graft survival in recipients with ECD kidneys (25). Searching for appropriate and effective biomarkers to assess renal quality and predict DGF is a topic of persistent interest in the field of kidney transplantation. Various scoring systems and biomarkers have been used to evaluate renal quality and predict DGF. However, they have certain limitations. Previous studies suggested that increased donor age remained an independent risk factor for DGF (26, 27). In contrast, a study by Helantera et al. was unable to demonstrate an association between donor age and DGF (28). Donor AKI or higher donor terminal Scr is associated with DGF (29). However, many factors can impact donor creatinine levels, including prerenal, intrarenal, and postrenal injury factors, which have limited the predictive value of donor terminal Scr for DGF (30, 31). The KDPI scoring system has been employed as an effective tool for evaluating the quality of donor kidneys, and a high KDPI typically has a stronger association with DGF and poorer transplant outcomes (32, 33). Due to the unique characteristics of this era, kidneys from ECD at extreme ages were utilized, which restricted the accuracy of the KDPI scoring system (34). Previous studies have demonstrated that the utilization of zero-point biopsy combined with the Remuzzi scoring system in guiding kidney transplantation is significant as it assists surgeons in accurately identifying the severity of AKI and chronic damage and provides a basis for early intervention and personalized treatment strategies to optimize patient outcomes (35, 36). However, the Remuzzi scoring system has limitations, including subjectivity in evaluation, lack of standardization across different centers, potential issues with specificity and sensitivity in identifying certain pathologies, failure to capture dynamic tissue changes, and reliance on the representativeness of biopsy samples, where certain types of damage may be missed due to insufficient sampling, which may lead to biased clinical decision-making. Additionally, the role of HLA mismatch in the short-term outcomes of kidney transplantation is subject to ongoing debate. While many studies indicate that lower HLA mismatches reduce the risk of acute rejection, leading to improved transplant success rates and kidney function recovery, other studies suggest that HLA compatibility is theoretically important but that its impact on actual clinical short-term outcomes may not be as significant as anticipated (37, 38). Our study found that the predictive value of donor urinary UDP-Glc for DGF was higher than that of other biomarkers. UDP-Glc is correlated with inflammation and injury (39). Additionally, the concentration of urinary UDP-Glc rapidly increases in the early stages of kidney injury, and extracellular UDP-Glc exhibits high stability (12, 40). The high concentration and stability of UDP-Glc provide a strong foundation for its use as a valuable biomarker for predicting DGF. UDP-Glc can provide transplant surgeons with a novel strategy to predict DGF earlier and more accurately without invasive procedures while also reducing medical costs.

Consequently, to further enhance the predictive value of the DGF, we combined clinical parameters to develop an effective predictive model. Notably, a combination of donor urinary UDP-Glc and donor terminal Scr, which demonstrated a relatively high predictive value for DGF in this study, was used to create the model. We found that the predictive value of the model improved, with an AUROC of 0.832 and a Youden index of 0.56. When donor urinary UDP-Glc, donor terminal Scr, and number of HLA mismatches were used to build a predictive model for DGF, the AUROC was 0.840, with a Youden index of 0.62. Additionally, when donor urinary UDP-Glc, donor terminal Scr, donor age, KDPI, donor Remuzzi pathology scores, and number of HLA mismatches were used to construct the model, the AUROC was 0.853, with a Youden index of 0.58. We considered these possibilities for the following reasons: first, utilizing multiple factors provides a comprehensive view of patient conditions and varied information that enhances decision-making capabilities; second, this approach reduces bias arising from single predictors, effectively captures complex relationships within the data, and improves generalization to unknown data. Additionally, advanced algorithms can leverage the integrated factors more efficiently, resulting in greater predictive accuracy. Overall, the integration of diverse clinical factors significantly boosts performance and clinical relevance (14, 41–44).

Finally, we showed that donor urinary UDP-Glc levels positively correlated with recipient Scr levels at 1 month after transplantation. This suggests that elevated urinary UDP-Glc concentrations in donors may reflect early kidney dysfunction in transplant recipients. Gaining a better understanding of the quality of donor kidneys, maintaining professional donor care, and effectively managing recipients can improve graft survival (45–47). However, our study had limitations that merit considering. These included a single-center observational clinical study design, a small sample size resulting in reduced statistical power, and a short follow-up period that restricted the assessment of long-term outcomes. Further research is needed to investigate the mechanisms involved and to evaluate the potential of donor urinary UDP-Glc as a predictive biomarker for long-term outcomes in kidney transplant recipients.

In conclusion, our study found that donor urinary UDP-Glc level was an independent risk factor for DGF. We believe that these findings can direct transplant surgeons towards novel strategies to predict DGF earlier and more accurately without invasive procedures while also reducing medical costs.

## Data Availability

The original contributions presented in the study are included in the article/**Supplementary Material**. Further inquiries can be directed to the corresponding author.
