# Supplementary material for: Preoperative donor urinary UDP-Glc as an independent risk factor for delayed graft function
Source: Front Immunol. 2025 Mar 17;16:1545280. doi: 10.3389/fimmu.2025.1545280 (PMC11955600; doi:10.3389/fimmu.2025.1545280)
Supplement: Supplementary file 1 [file DataSheet1.docx]

Supplementary Material

# Supplementary Figures and Tables

## Supplementary Figures


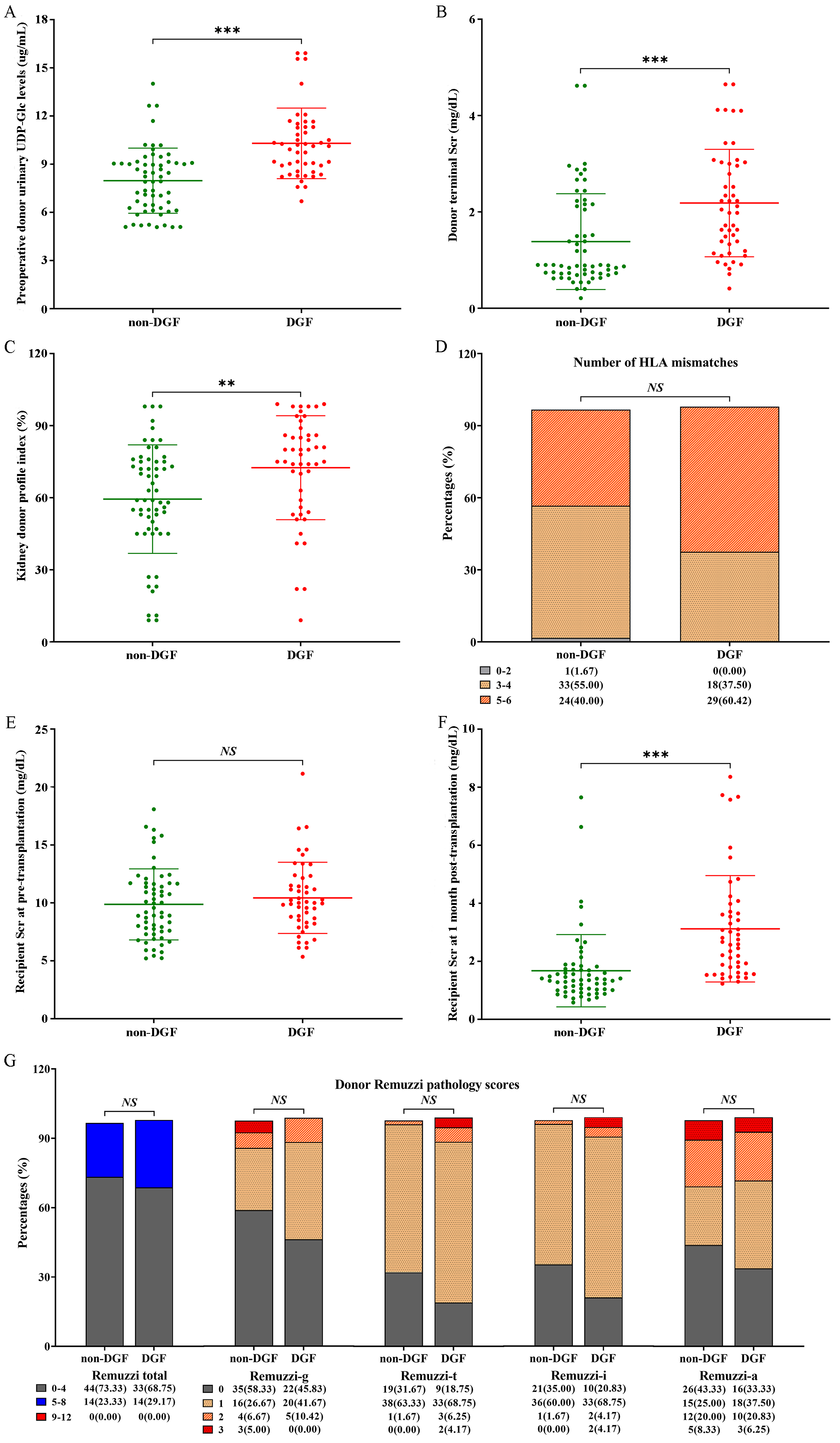


**Supplementary Figure S1.** Distribution of several relevant clinical factors in the DGF and non-DGF subgroups. (**A**) Preoperative donor urinary UDP-Glc levels; (**B**) donor terminal Scr; (**C**) kidney donor profile index; (**D**) number of HLA mismatches; (**E**) recipient Scr at pre-transplantation; (**F**) recipient Scr at 1 month post-transplantation; (**G**) donor Remuzzi pathology scores. DGF, delayed graft function; UDP-Glc, Uridine diphosphate glucose; Scr, serum creatinine; HLA, human leukocyte antigen; g, glomerular global sclerosis; t, tubular atrophy; i, interstitial fibrosis; a, arterial and arteriolar narrowing. *NS,* non-significant; ***p* < 0.01; ****p* < 0.001.
